# Origin of observed narrow bandgap of mica nanosheets

**DOI:** 10.1038/s41598-022-06820-5

**Published:** 2022-02-21

**Authors:** Shunnian Wu, W. P. Cathie Lee, Ping Wu

**Affiliations:** Entropic Interface Group, Engineering Product Development, Singapore University of Technology and Design, 8 Somapah Road, Singapore, 487372 Singapore

**Keywords:** Chemistry, Materials science, Nanoscience and technology

## Abstract

Mica nanosheets possess peculiar feature of narrowed bandgap with the decrease of thickness but a conclusive theoretical understanding of the narrowing mechanisms is still under development. In this report, first-principles calculations were carried out to investigate the electronic band structure of mica nanosheets with the deposition of K_2_CO_3_. Bulk mica shows an indirect bandgap of 4.90 eV. Mica nanosheets show similar electronic structures to bulk mica with a gradually increased bandgap of 4.44 eV, 4.52 eV and 4.67 eV for 1-layer, 2-layers and 3-layers nanosheets, respectively, which is attributed to the lattice relaxation. K_2_CO_3_ is found to have strong affinity towards mica nanosheets. The K_2_CO_3_ deposited mica nanosheets showed an increased bandgap with the increase of thickness, consistent with experimental observations. The calculated bandgap of K_2_CO_3_ deposited mica for 2-layers and 3-layers nanosheets are 2.60 eV and 2.75 eV, respectively, which are comparable with the corresponding experimental values of 2.5 eV and 3.0 eV. Our theoretical findings support the experimental evidence of surface contamination of mica by K_2_CO_3_, and provide new insight into the structure and properties of 2D mica.

## Introduction

Mica is a naturally occurring sheet-like silicate mineral important in rock-formation, whose crystal structure is typically associated with the chemical formula of KAl_2_(Si_3_Al)O_10_(OH)_2_. It possesses a series of impressive properties, including visible-light transparency, ultraviolet (UV)-shielding, atomic level flatness, electric insulation, temperature stability, and chemical durability^1^. Therefore, mica has a wide range of applications, ranging from insulation of electronic components to an ingredient in cosmetics. The versatility of mica attracts researchers to explore its potential applications including the formation of self-assembled organothiol monolayer as a template for crystalline growth^2^, preparation of mica glass–ceramics materials for hydrogen storage^3^, growth of flexible epitaxial ferroelectric oxide thin films for piezoelectric energy harvesting^4^, synthesis of ZnO nanorods for dye-sensitized solar cells and piezoelectric nanogenerators^5^, and fabrication of flexible mica film for high-temperature energy storage^6^.

Recently, the interest in 2D nanosheets of mica has emerged with the encouraging progress in its preparation techniques, as mica can be cleaved to yield an atomically flat surface since its layered aluminosilicate sheets are bound to alternate layers of K^+^ ions. Four main approaches have been developed: (1) Mechanical exfoliation technique. Mica is reported to be exfoliated down to few mono-layers by mechanical exfoliation using sticky tape technique^7^. Besides that, atomically thin mica sheets ranging from 14 to 2 layers were also obtained by mechanical exfoliation using polydimethylsiloxane (PDMS) stamps^8^. (2) Sonication exfoliation. Mica nanosheets were obtained by applying a sonication technique to bulk micas, where the thickness of the mica sheets are dependent on the sonication duration^9^. (3) Intercalation-promoted exfoliation technique. Weak interlayer forces facilitate the exfoliation of mica^10^. Intercalation between interlayers increases the basal spacing and decreases the attraction force between layers of mica. Stable monolayer mica was prepared through intercalation by octadecyl trimethyl ammonium chloride (OTAC) followed by micromechanical ultrasonic cleavage to nanosheets^10,11^. Large-scale exfoliation of mica into mono- or few-layered mica nanosheets was achieved with ultrasonication exfoliation after intercalation by cetyltrimethylammonium bromide (CTAB)^1^. High-quality mica nanosheets were exfoliated from natural ground mica after a combination of CTAB intercalation and ultrasonication treatment^12^. Amphipathic poly(vinylpyrrolidone) (PVP) polymer can prevent self-aggregation of exfoliated nanosheets through the repulsive forces stemming from hydrophobic polyvinyl chains that stretch towards solvents, further overcome the difficulties in exfoliations of large area nanosheets from bulk mica without the application of ultrasonication process^13^. (4) Microwave-expanded exfoliation technique. With the advantage of rapid heating, microwave irradiated expansion integrated with solvothermal method is effective to exfoliate mica into single- and few-layer nanosheets with mild sonication^14,15^. The nanosheets further improve the potential applications of mica and extend the application field. Furthermore, the mechanical properties of mica nanosheets (i.e. low pre-tension and high Young’s modulus and breaking force) validate their applicability in highly demanding mechanical applications such as flexible ultrathin insulating substrates/dielectrics or for reinforcement in nanocomposites^8^. A mica membrane was constructed by regularly stacking mica nanosheets and then immobilized with ionic liquid (IL) into its 2D channels to separate CO_2_ from H_2_, CH_4_ and N_2_^12^. Potential application in photocatalytic degradation of dyes was recently demonstrated by our research group.

Particularly, mica nanosheets are found to show reduced bandgap corresponding to semiconductor regime. Bae et al. reported a measured optical bandgap of 4.13 eV^13^, which was considerably smaller than that of bulk mica (about 7.85 eV)^16^. Exfoliated mica has a downward bandgap of 4.40 eV, 3.91 eV, and 3.62 eV with the enhanced degree of exfoliation, though the layer numbers were not identified^9^. It was claimed to be attributed to the surface effect and/or lattice relaxation with regard to atomic reconfiguration and re-coordination^9^. Furthermore, the bilayer case exhibits a semiconducting nature with the measured bandgap of ∼ 2.5 eV^15^. The bandgap narrowing was proposed to be the consequence of lattice relaxations and surface doping effects^15^. However, the origin of the bandgap narrowing is not clarified.

It is known that preparing mica surfaces that are truly clean is not easy since mica has a high-energy surface that readily adsorbs water, organic contaminants, and gases from the atmosphere. A freshly cleaved mica surface contains large number of active sites which reacts chemically with the environment, thereby changing its physicochemical properties^17^. Mica can also become charged during cleaving, which makes it prone to pick up oppositely charged particles or mica flakes from the surroundings^18^. Unless they are cleaved and maintained in ultrahigh vacuum, it is relatively impossible to avoid adsorption of some foreign substance on any “clean” (high-energy) solid surfaces.

Previous experiments have observed the growth of potassium carbonate (K_2_CO_3_) crystals on the exposed mica surface due to the reaction of water and CO_2_ with K^+^ ions^18–20^, although the details of the reaction leading to the formation of K_2_CO_3_ on mica are yet to be clarified^21^. In addition, C atomic concentration is found to be slightly increased during exfoliation, presumably due to the contamination of the exposed mica surface^14^. It is equally certain that the K_2_CO_3_ can have no effect on the properties of the mica surface after it is immersed in bulk water or aqueous solution, thereby dissolving the K_2_CO_3_.

Thus, a detailed understanding of the origin of the narrowed bandgap of mica nanosheets is important for accurate description of their structural and electronic properties. This is crucial for their potential electronic and photonic applications.

## Results and discussion

### Structural and electronic properties of bulk mica

**Figure 1:**
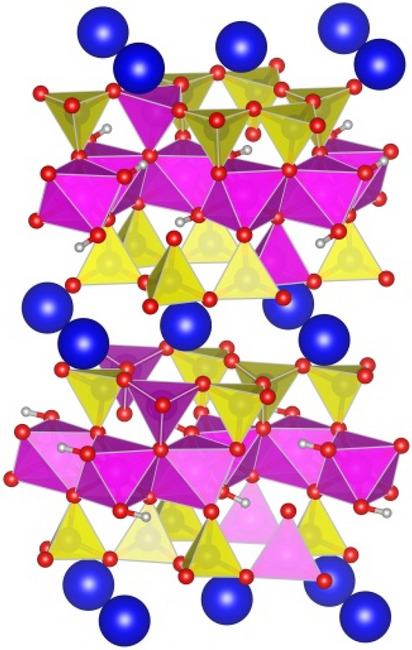
The structure of optimized bulk mica. Colored atoms are shown as: H (grey), O (red), K (blue), Al (pink) and Si (yellow). The coordination polyhedra around the Al atoms (pink polyhedra) and Si atoms (yellow polyhedra) are shown as well.

Mica consist of tetrahedral and octahedral layers stacked in a 2:1 ratio and bonded by interlayer K^+^ cations with the formula KAl_2_(AlSi_3_O_10_)(OH)_2_^22^. The Si^4+^ cations in the tetrahedral layers are replaced by Al^3+^ at a ratio of 3:1, which is charge balanced by K^+^ cations intercalated between the tri-layers. The stable structure identified by Militzer et al.^23^ was used as the starting configuration in this work. Therefore, the smallest repeat unit adopted for bulk mica calculations is the 1 × 1 × 2 supercell containing 84 atoms. Our preliminary calculations indicate that placing two Al^3+^ cations in the same tetrahedral layer increases the total energy by more than 3.5 eV. Complying with the Loewenstein Al avoidance rule^24^, we set the optimization criteria as maximizing the distance between the Al^3+^ sites to minimize the Ewald energy. The most stable configuration was obtained after performing full geometry optimization with imposing the constriction of inversion symmetry. It is noted that the energy differences is relatively small (less than 0.15 eV per unit cell) between configurations of permutation of one Al^3+^ site in the same layer. The mica structure is shown in Fig. 1. It can be seen that four tetrahedral Al^3+^ atoms are located in different tetrahedral layers with a maximum separation distances.

**Figure 2:**
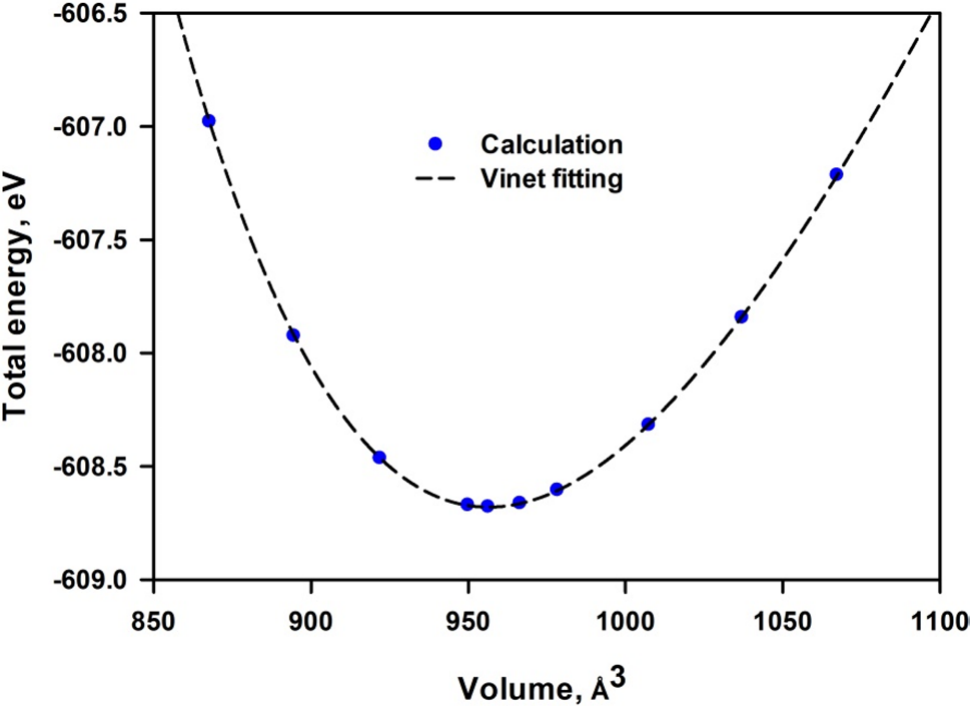
Variation of total energy with volume fitted with the Vinet equation.

To determine the cell volume of the most stable mica, the total energy (E) at various volume (V) is calculated as shown in Fig. 2. The Vinet equation of states is asserted to be more suited for flexible structures^25,26^. Since mica possesses great flexibility with significant anharmonicity, we employed the Vinet equation, which takes the following form^27,28^:

1 $$ E\left( V \right) = E_{min} + \frac{{2B_{0} V_{min} }}{{\left( {B_{0}^{\prime } - 1} \right)^{2} }} \left[ {2 - \left( {5 + 3B_{0}^{\prime } \left( {\lambda - 1} \right) - 3\lambda } \right) \times exp\left( { - \frac{3}{2}\left( {B_{0}^{\prime } - 1} \right)\left( {\lambda - 1} \right)} \right)} \right],\quad {\text{where}}\;{{\uplambda = }}\sqrt[3]{{\frac{V}{{V_{min} }}}} $$

where E_min_ and V_min_ are the minimum energy and the corresponding volume, respectively; B_0_ is the bulk modulus, and $B_{0}^{\prime } = \frac{{\partial B_{0} }}{\partial P}$ is the derivative of the bulk modulus with respect to pressure. It is noted that the calculated bulk modulus represents the response of the system under isotropic compression.

It is seen from Fig. 2 that the relation between total energy and volume agrees well with the Vinet equation. The obtained V_min_ and B_0_ are listed in Table 1. The calculated volume 956.47 Å^3^ shows less than 4% deviation with the experimental measurement. The bulk modulus is also in good agreement with the experimental results. It is noted that the experimental structural data in Table 1 exhibit much discrepancy, which can be contributed to differences in the content of impurities in the mica samples used in different experiments. Therefore, the density functional theory (DFT) calculations can provide sufficient description of mica structure.

**Table 1:** Structural data of bulk mica.

	Cal	Exp^29^	Exp^30^	Exp^31^
a (Å)	5.22	5.19	5.16	5.23
b (Å)	9.05	9.01	8.90	9.07
c (Å)	20.36	20.06	20.07	20.04
β (°)	95.33	95.80	95.75	95.74
V_min_ (Å^3^)	956.47	934.40	921.90	945.40
B_0_ (GPa)	50.1	49.0	67.7	66.5

**Figure 3:**
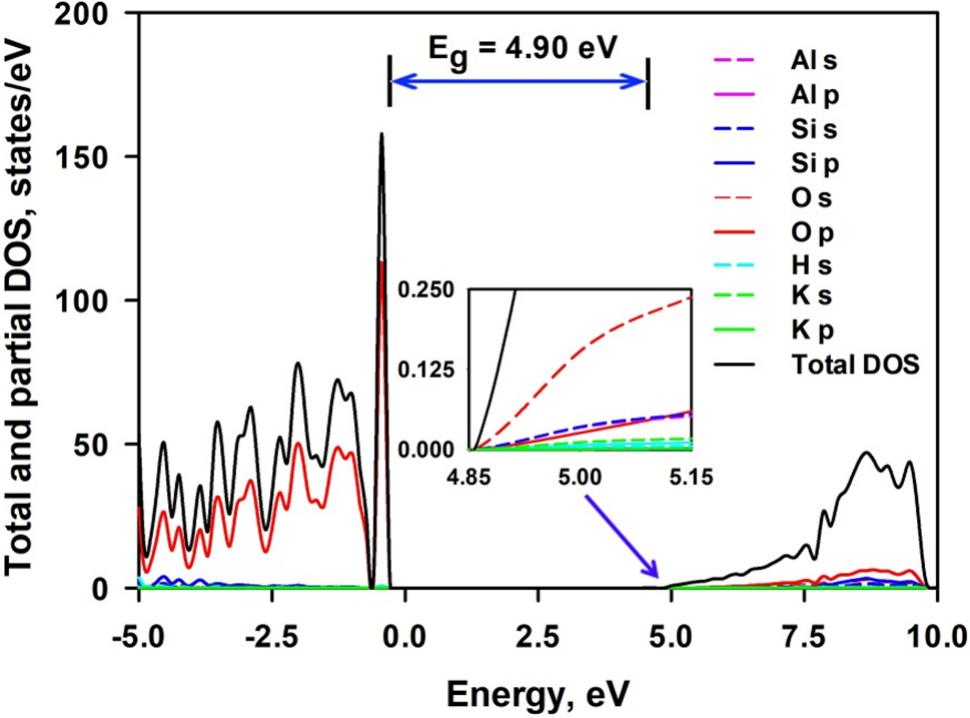
Total and projected density of electronic states of bulk mica. The inset is a magnificence of the CBM region. The energy is given relative to the Fermi energy set at zero.

Figure 3 shows the total and projected density of electronic states (DOS) of bulk mica. The valence band was composed of s orbital of H, s and p orbitals of K, Al and Si, and p orbital of O, while the conduction band is composed of s orbital of Si, H, K and O and p orbital of O, Si and K. Hybridization between O (2*p*) states and states from Si, Al and H is observed. The valence band maximum (VBM) is formed by the 2*p* electrons of O atoms, and the contribution from all other atoms to the VBM is negligible. The conduction band minimum (CBM) is derived from the predominant s orbitals of O atoms and slight contribution from s orbitals of Al atoms and Si atoms. It appears that O atoms play controlling role in the electronic structure of mica. It is noted that the interlayer K cations play insignificant role in the electronic structure, since the major peaks originated from the K states are located at about − 10 eV, far away from the Fermi level. Therefore, a substitution of interlayer K cation should have no influence on the bandgap. This indicates that it may be tough to tailor the bandgap energy of mica through ionic exchange of interlayer cations.

**Figure 4:**
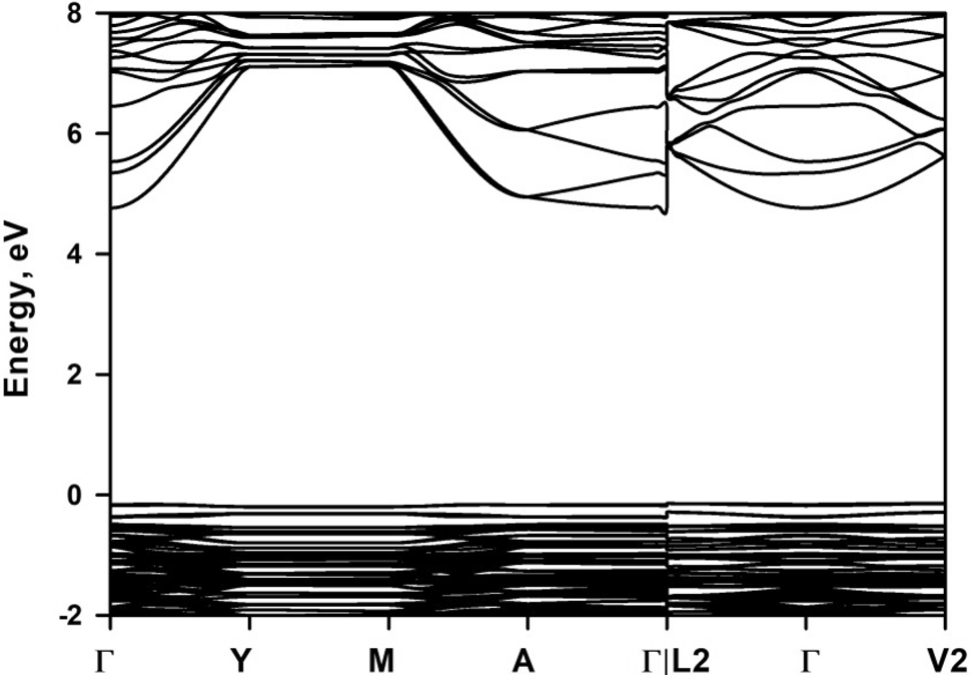
Band structure of bulk mica along high symmetry points.

Figure 4 shows the band structure of mica along some high symmetry points. It is seen that CBM is located at gamma point at the energy level of 4.76 eV, while VBM is located close to V2 point at the energy level of − 0.14 eV. This gives rise to an indirect bandgap of 4.90 eV. This value is in agreement with the theoretical report of 4.83 eV by Zheng et al. without Van der Waals correction^32^, and 4.82 eV by Vatti et al. with D2 correction method of Grimme et al.^33^ for Van der Waals interaction^34^. It seems that Van der Waals correction has unsubstantial effect on bandgap. However, Kim et al. reported a much smaller calculated bandgap energy of 3.16 eV^15^. It is supposed that they employed first-order approximation of mica structure without any Al^3+^ substitution of Si^4+^, which can be derived from the substantial contribution of Si electrons while having inappreciable contribution of Al electrons to conduction band as shown in their density of electronic states curve of bulk mica. On the other hand, experimental bandgap values with great discrepancy are reported in the literature. Davidson et al. claimed that mica is an insulator material with a large bandgap of 7.85 eV^16^. Kaur et al. determined the bandgap of the mica to be 3.4 eV^35^. Thermoluminescence experiments by Kalita et al. derived the bandgap to be 5.09 eV^36^, while diffuse reflectance spectrum measurement generates an optical bandgap of ∼ 4.65^7^. Thus it is difficult to make comparison between our calculations and experimental work. However, it is noted that DFT calculations generally underestimate the bandgap value^37^. A hybrid functional can achieve the best possible description for the bandgap if the computation cost can be dealt with^38^. A bandgap of 6.83 eV is obtained with use of the hybrid functional PBE0 and setting the α = 0.25 to account for the screening properties^34^.

### Mica layer structure

**Figure 5:**
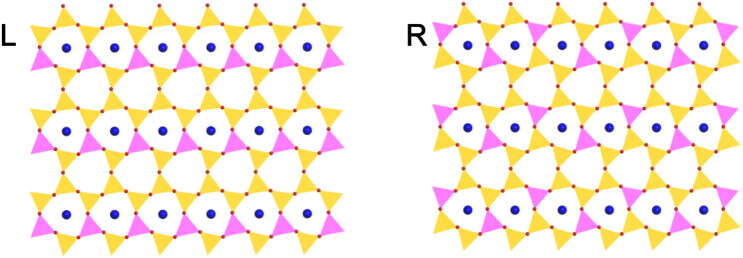
Expanded unit cell of one-layer mica cleaved from bulk mica (**L**) and that with applied shift of tetrahedral Al^3+^ atom (**R**). Atoms are shown as colored balls: H (grey), O (red), K (blue), Si (yellow) and Al (pink). The coordination polyhedra around the Si atoms (yellow polyhedra) and Al atoms (pink polyhedra) are shown as well.

We carried out the first-principles calculations to understand the structural and electronic properties of mica layers, including 1-layer, 2-layers and 3-layers. DFT calculation of 4-layers mica is too expensive to our research group currently. Since we employed 84-atom unit cell for bulk mica, it gave rise to a 42-atom repeat unit cell of 1-layer mica. This gives us an opportunity to expand the repeat unit cell for 1-layer mica in a more stable configuration. Figure 5L shows the expanded repeat unit cell of 1-layer mica cleaved from bulk mica. It can be seen that the distance between the tetrahedral Al^3+^ atoms is not maximized to reduce the repulsion force. This indicates that a more favorable distribution of tetrahedral Al^3+^ atoms can be obtained if each tetrahedral Al^3+^ ions is shifted to the opposite of the hexagonal rings to reduce the Al^3+^-Al^3+^ repulsion interactions. The configuration after the Al^3+^ shift is shown in Fig. 5R. The total energy calculation indicates that there exist an energy difference of 0.25 eV per unit cell, and the configuration shown in Fig. 5R is more stable. Monte Carlo simulations using larger super cells, with up to 36 substitution sites, observed the same arrangement of cations to be most favorable^39^. However, due to the large number of atoms involved in the unit cell of 2-layers and 3-layers mica, we adopted the unit cell cleaved from bulk mica structure.

**Figure 6:**
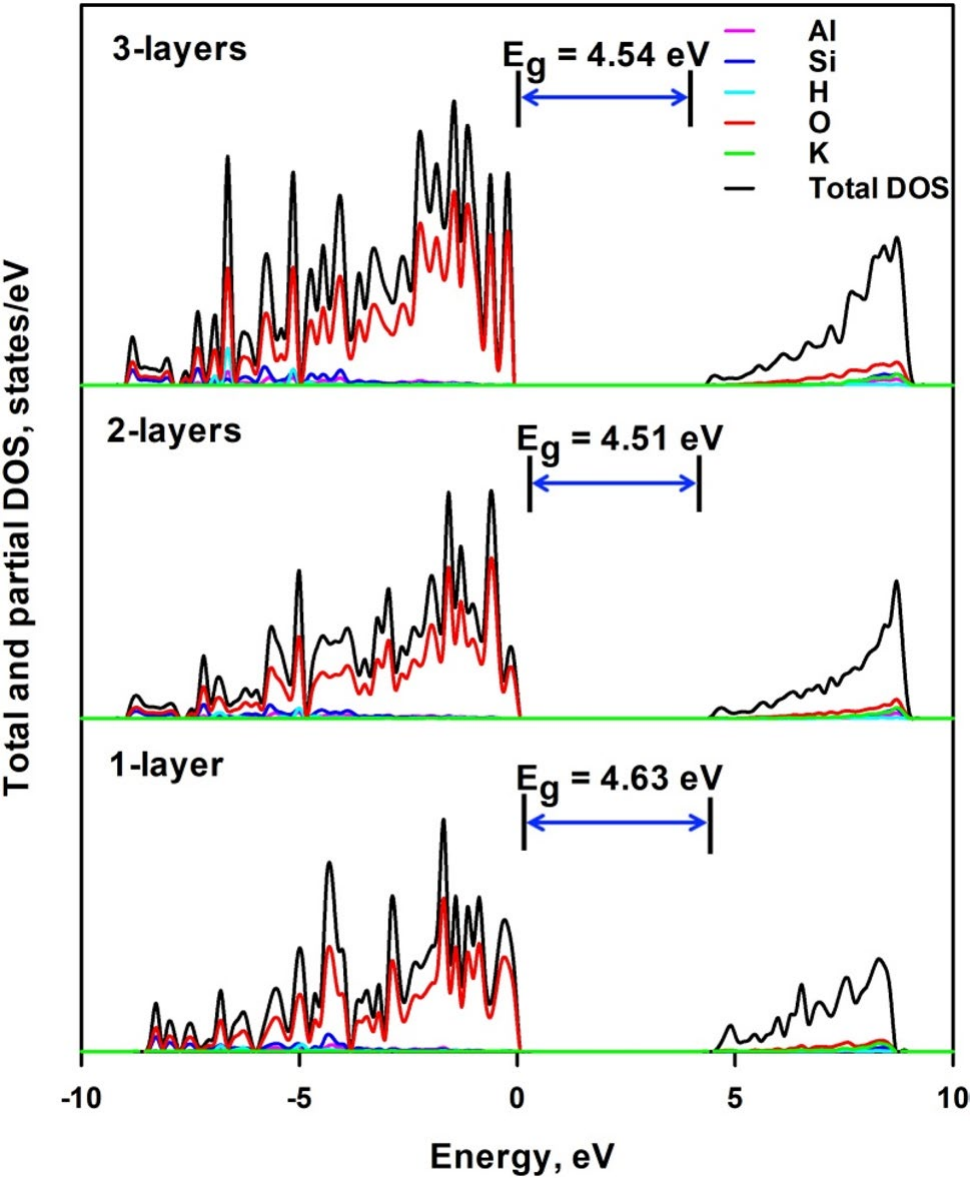
Total and projected density of electronic states of 1-layer, 2-layers and 3-layers mica.

Figure 6 shows the total and projected density of electronic states of 1-layer, 2-layers and 3-layers mica. It is seen that the general electronic structure features were similar to that of the bulk mica. Although the VBM is still formed by the 2*p* electrons of O atoms, K cations make significantly increased contribution to the conduction band, and the CBM comes from orbitals from O, K, Si and Al atoms. The relative contribution of K cations is increased with the decrease of the layer numbers. CBM is observed to move closer to the fermi level with the decrease of thickness. This brings about an increase of bandgap energy with the thickness of mica layers, i.e. 4.44 eV (for the less stable 1-layer structure), 4.51 eV and 4.54 eV for 1-layer, 2-layers and 3-layers mica, respectively. The more stable 1-layer mica nanosheet shows a larger bandgap of 4.63 eV than the less stable structure, which may implicate that the calculated bandgap for 2-layers and 3-layers mica nanosheets will be underestimated. Park et al.^9^ also reported an increasing trend of bandgap energy from 3.62 to 4.40 eV with the increase of mica layers. Using artificial intelligence techniques and neural network models, bandgap energy of semiconductors^40^ is disclosed to be dependent on the competition of attractive and repulsive forces, which can be represented by valence electron transfer and shortest interatomic distance. Most nanomaterials with diameters of about 2–10 nm show an increased bandgap compared with the bulk semiconductor due to the quantum confinement effects^41–46^, where the change in valence electron transfer plays a central role. On the other hand, lattice relaxation effect to cause a change in shortest interatomic distance, acts as competition to reduce bandgap energy in some nanomaterials^47^. Table 2 shows the variation of lattice parameters with the number of mica layers; here c parameters are defined as the averaged distance of interlay K cation planes in the vertical *z* direction. It is seen that the lattice parameters increase with the decreased layer numbers of mica nanosheets, indicating enlarged interatomic distance. Therefore, the 1-layer mica nanosheet has the largest interatomic distance, while the bulk mica has the lowest interatomic distance. This will lead to the bandgap narrowing in mica nanosheets compared with bulk mica. Table 3 shows the variation of the average Bader charge of each atom with the layer numbers of nanosheets, which is employed to evaluate the valence electron transfer. Both electron donations by Al, Si and K and electron acceptance by O increase remarkably from bulk mica to mica nanosheets. This increases the layer-layer electrostatic interaction from bulk mica to mica nanosheets. The electron donation of K atom among three mica nanosheets shows insignificant variation, which may suggest its major role for charge compensation. Though the electron donation by H atom is decreased with the increased layer numbers of nanosheets, H–O bonds do not affect the layer-layer electrostatic interaction since they are located in the *xy* plane rather than the vertical *z* direction. The increase of electron acceptance by O atom is supposed to increase ionic bonding in mica nanosheets, which generally tends to increase bandgap energy^48^. Therefore, both lattice relaxation effect and quantum confinement effects occur in the mica nanosheets, and lattice relaxation effect plays the predominant role in narrowing the bandgap energy. The lattice relaxation effect is also observed by Kim et al.^15^ though the mica structure they adopted is different.

**Table 2:** Calculated structural data of mica nanosheets.

	1-layer	2-layers	3-layers
a (Å)	5.29	5.26	5.23
b (Å)	9.10	9.07	9.06
c (Å)	12.28	12.16	11.77
β (°)	95.98	95.41	95.38

**Table 3:** Bader charge of atoms.

Atom	1-layer	2-layers	3-layers	Bulk
Al	− 3.525	− 3.525	− 2.475	− 2.235
Si	− 3.164	− 3.158	− 3.157	− 2.897
H	− 0.606	− 0.607	− 0.613	− 0.633
O	1.585	1.584	1.583	1.440
K	− 0.889	− 0.889	− 0.889	− 0.615

Our results are in consistence with the lower bandgap energy of ~ 5.7 eV for a single-layer mica than the bulk material (~ 7.8 eV) calculated by Gao et al. with band calibration using experimental data^49^. A comparison of bandgap between monolayer and bulk Mg(OH)_2_ and between monolayer and bulk Ca(OH)_2_ leads to the similar observation. For monolayers, the bandgaps are 4.80 (3.30) eV for Mg(OH)_2_ and 5.16 (3.68) eV for Ca(OH)_2_ from HSE06 (PBE), while for bulk materials, the bandgap values are 6.37 (4.59) eV for Mg(OH)_2_ and 6.12 (4.37) eV for Ca(OH)_2_ from HSE06 (PBE)^50^. This contradicts the substantial reduction of calculated bandgap values for mica nanosheets by Kim et al.^15^ This may be due to their first-order approximation of parent mica structure without Al^3+^ replacement. The experimental bandgap energies of mica nanosheets of 2, 3, and 4 layers are reported to be 2.5 eV, 3.0 eV and 3.4 eV, respectively^15^. This suggests that the observed bandgap narrowing with regard to bulk mica may be due to certain unnoticed mechanism, probably surface contamination of mica nanosheets by K_2_CO_3_.

### Mica-K_2_CO_3_ composite structure

K_2_CO_3_ crystal is of the same hexagonal space group with mica, and moreover, its lattice parameters a = 5.64 Å, b = 9.80 Å, c = 6.88 Å and β = 98.81° match with those of mica^51^.

**Figure 7:**
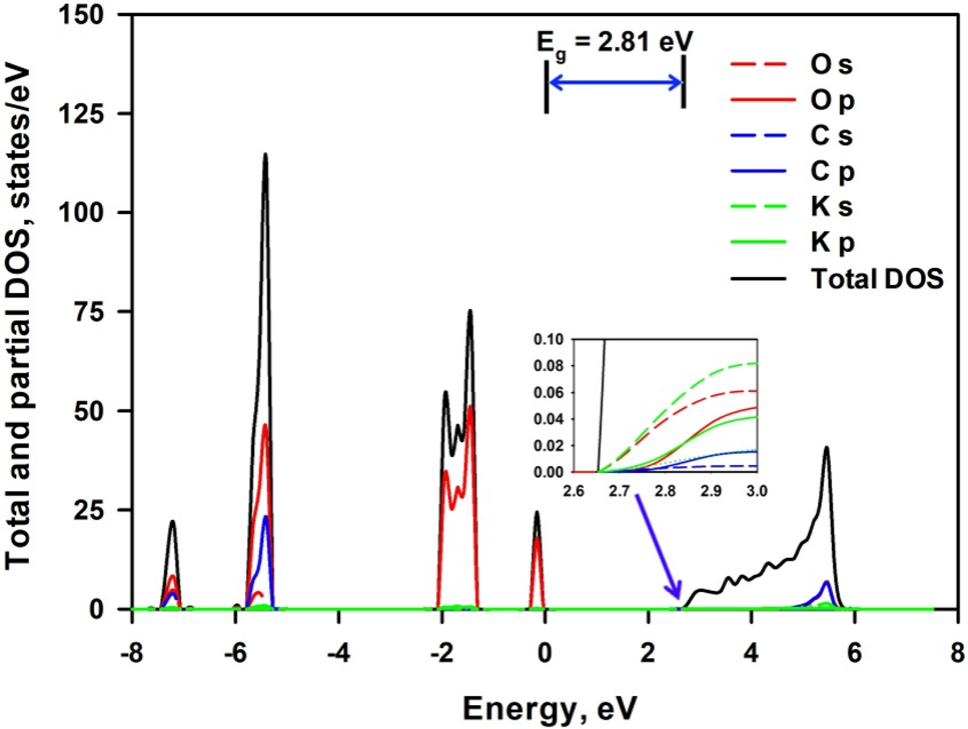
Total and projected density of electronic states of 1-layer K_2_CO_3_. The inset is a magnificence of the CBM region. The energy is given relative to the Fermi energy set at zero.

K_2_CO_3_ is an insulator material with a theoretical reported bandgap of 3.70 eV^52^. Figure 7 shows the calculated total and projected density of electronic states of 1-larer K_2_CO_3_. It can be seen that the VBM is contributed by O 2*p* states, while the CBM are mainly composed of K 4*s* states and O 2*s* states. A bandgap of 2.94 eV is obtained from Fig. 7, which is smaller than 3.75 eV of bulk K_2_CO_3_. The calculated lattice parameters of 1-layer K_2_CO_3_ are a = 5.58 Å, b = 9.56 Å, c = 6.88 Å and β = 97.32°, while the calculated lattice parameters of bulk K_2_CO_3_ are a = 5.76 Å, b = 9.90 Å, c = 7.19 Å and β = 97.32°. This indicates that the reduced bandgap energy of 1-layer K_2_CO_3_ with regard to bulk K_2_CO_3_ is not due to the lattice relaxation. It is observed that the contribution of K atoms to the conduction band is significantly increased in 1-layer K_2_CO_3_, therefore, the reduction in bandgap energy seems to be owing to the enhanced electron transfer from K atoms to O atoms to facilitate the formation of unoccupied 4*s* orbitals of K atoms.

**Figure 8:**
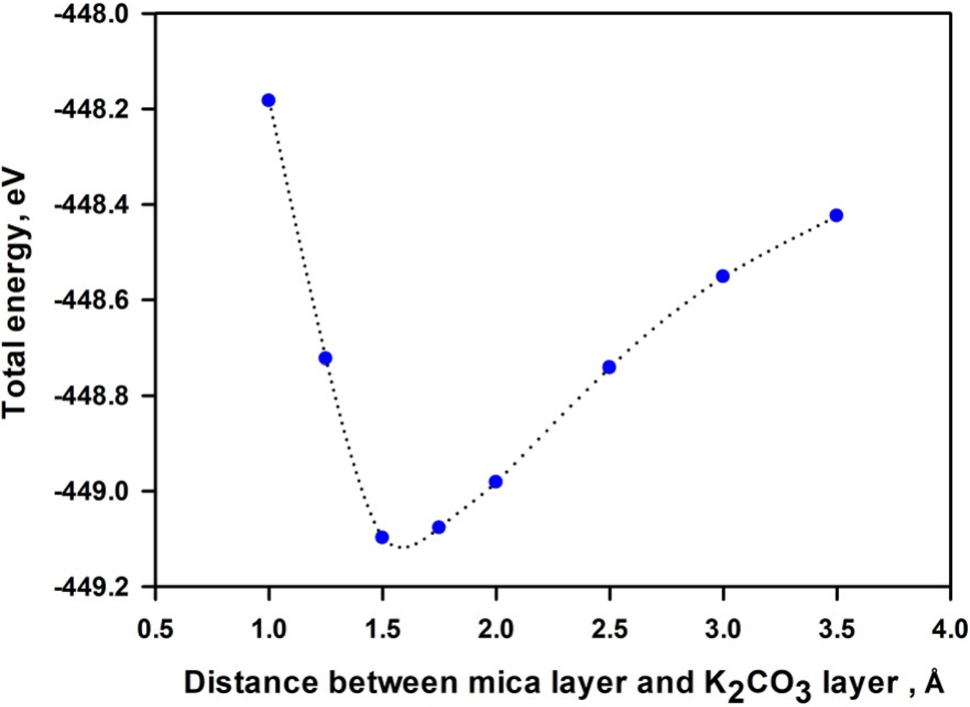
Variation of total energy with interlayer distance between 1-layer mica and 1-layer K_2_CO_3_. Interface distance between 1-layer mica and 1-layer K_2_CO_3_ is represented by the difference between averaged z coordinates of atoms in the bottom-most of K_2_CO_3_ and in the top-most of mica.

Figure 8 shows the variation of the total energy with the interlayer distance between deposited 1-layer K_2_CO_3_ and 1-layer mica. When the interlayer distance is below 1.5 Å, repulsion interaction dominates. The system reaches the most stable configuration at the interlayer distance of ~ 1.55 Å. Hence, our calculations of electronic structure of mica nanosheets—K_2_CO_3_ composites adopt the interlayer distance of 1.55 Å. Our testing indicates that a slight change of the interlayer distance does not lead to appreciable variation of bandgap energy. Further increase in the interlayer distance weakens the attraction interaction between K_2_CO_3_ and mica, thus the total energy gradually increases. The calculated binding energy between K_2_CO_3_ and 1-layer, 2-layers and 3-layers mica nanosheets are − 3.40 eV, − 3.59 eV and − 3.48 eV respectively. The negative values indicate that the binding between K_2_CO_3_ and mica is thermodynamic stable. The lower binding energy for 1-layer mica may be due to the fact that the more stable 2 × 1 × 1 unit cell was used for 1-layer mica, which is different from the unit cell for 2-layer and 3-layer mica. The less stable 1-layer nanosheet would give a binding energy of − 3.64 eV. Therefore, the affinity between K_2_CO_3_ and mica nanosheets increases with the decrease of layer number. Therefore, the affinity between K_2_CO_3_ and bulk mica is supposed to be the weakest.

**Figure 9:**
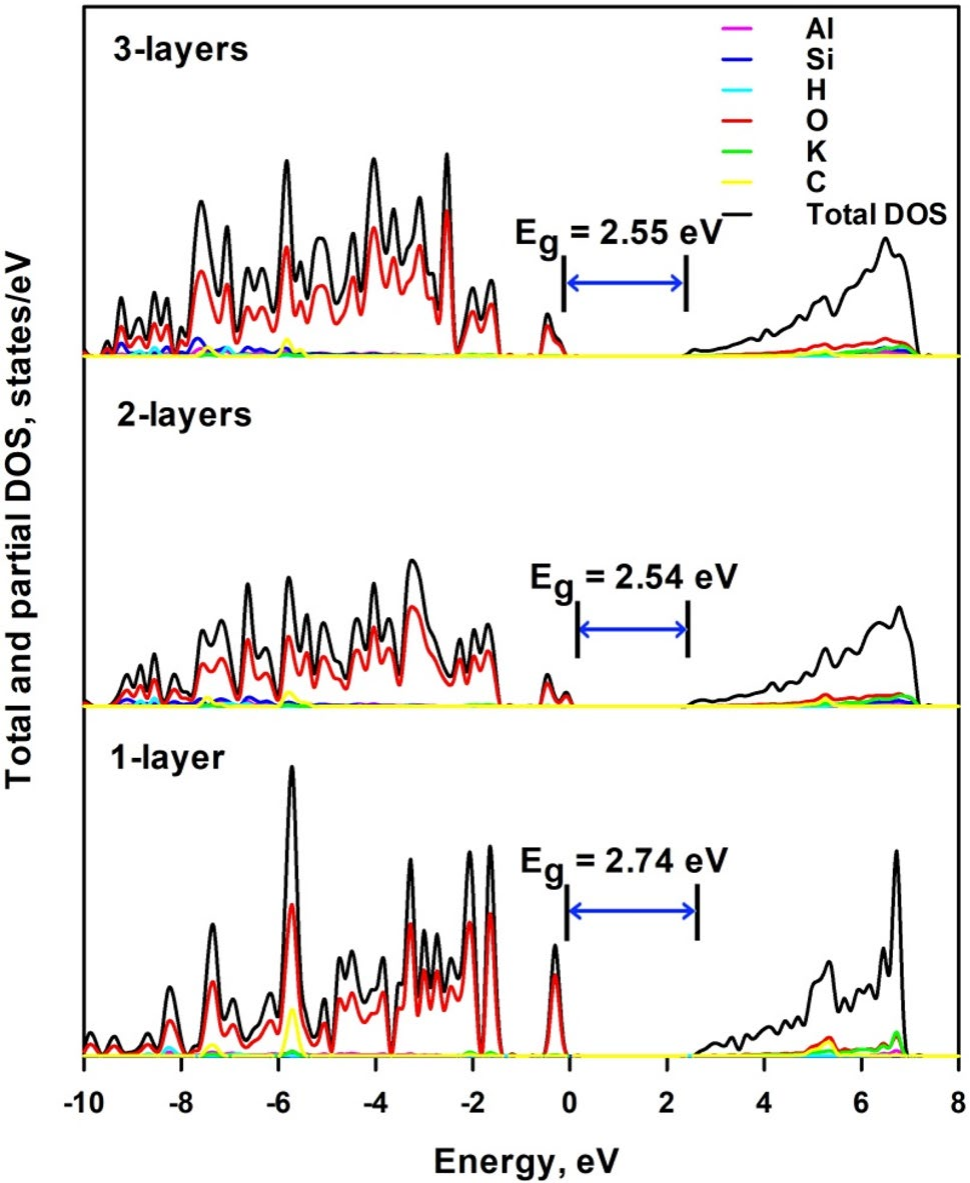
Total and projected density of electronic states of mica nanosheets-K_2_CO_3_ composite structure.

Figure 9 shows the total and projected density of electronic states of mica nanosheets—K_2_CO_3_ composite structure. 1-layer K_2_CO_3_ is deposited on 1-layer, 2-layers and 3-layers mica. Similarly to mica nanosheets, the VBM of the composite is formed by the 2*p* electrons of O atoms; however the C atoms make even greater contribution than K atoms to conduction band though the highest contribution is still from 2*s* electrons of O atoms. It is seen from Fig. 9 that the bandgap energy is 2.74 eV (2.47 eV for the less stable 1-layer structure), 2.54 eV and 2.55 eV for 1-layer, 2-layers and 3-layers mica, respectively. It increases with the number of mica layers, which is also due to the lattice relaxation. The deposition of 1-layer K_2_CO_3_ significantly reduces the corresponding bandgap energy of mica nanosheets. This may be due to the considerable charge transfer from C atoms and K atoms to O atoms, which is beneficial to the generation of unoccupied 2*p* orbitals in C atoms and 4*s* orbitals of K atoms. This lowers the energy level of CBM and thus reduces the bandgap energy. A detailed analysis of the charge transfer deserves further experimental and theoretical studies. The bandgap energies of mica nanosheets for 2- and 3-layers via experiments are 2.5 eV and 3.0 eV, respectively^15^. These values agree well with our calculations. The correspondence between theoretical results and experimental data allows us to conclude that the deposited K_2_CO_3_ should be the origin of the observed tunable bandgap in layered mica^15^. Since the affinity between mica nanosheets and K_2_CO_3_ decreases with the increase of thickness of mica nanosheets, the effect of bandgap narrowing would become weaker with the increase of layer numbers.

Since surface contamination by K_2_CO_3_ is dependent on the mica cleavage techniques^18–20^, different preparation techniques would generate mica nanosheets with discrepant degree of K_2_CO_3_ deposition. Mica sheets prepared by tape-cleaving technique probably produces the cleanest surfaces^18–20^, therefore, mica nanosheets prepared by mechanical exfoliation technique would demonstrate the least K_2_CO_3_ deposition. This suggests that nanosheets bandgap may also be tailored by preparation techniques.

The appearance of K_2_CO_3_ significantly affects the crystal structure and electronic properties of the yielded mica nanosheets. Though this may not bring about appreciable difference to its application in aqueous solutions, since the deposited K_2_CO_3_ dissolves (solution pH may be fluctuated), it will greatly shift its potential application in solid states, such as component of semiconductor or photocatalyst or energy storage, which deserves further experimental and theoretical research.

In summary, the exfoliation of bulk mica to 1-layer nanosheet reduces the bandgap by about 10% from 4.90 to 4.44 eV. Deposition of K_2_CO_3_ on mica nanosheets further dramatically decreases the bandgap energy, for example, the 1-layer nanosheet significantly lowers its bandgap by about 45% from 4.44 to 2.47 eV. Our results indicate that not a single factor solely determines the bandgap energy of mica nanosheets. Both lattice relaxation effect and quantum confinement effects occur in the mica nanosheets. Lattice relaxation will bring about the increase of the shortest interatomic distance, which leads to a narrowed bandgap energy, while quantum confinement will change the layer-layer interaction with an alteration of valence electron donation and acceptance of each atom, which tends to increase the bandgap energy. The results indicate that lattice relaxation plays the dominant role in controlling the bandgap energy.

## Conclusion

The electronic structures of bulk mica, mica nanosheets, and K_2_CO_3_–deposited mica nanosheets were obtained using first-principles calculations in this study. Bulk mica shows an indirect bandgap of 4.90 eV, with the VBM formed by O 2*p* states and the CBM derived from the dominant O 2*s* states. Mica nanosheets show similar electronic structures to the bulk mica but significantly increased contribution to conduction band by K cations. A gradually increased bandgap of 4.44 eV, 4.52 eV and 4.67 eV is observed for 1-layer, 2-layers and 3-layers mica nanosheets, respective, which is due to the lattice relaxation. K_2_CO_3_ shows strong affinity with mica nanosheets. and 1-layer K_2_CO_3_ manifests an increased affinity with the decrease of layer number of mica nanosheets. The K_2_CO_3_-deposited mica nanosheets show increased bandgap energy with the increase of thickness, and the calculated bandgap values for 2-layers and 3-layers mica are 2.54 eV and 2.55 eV, respectively, which are consistent with the experimental reported 2.5 eV and 3.0 eV separately. Our results give theoretical support to experimental proposed surface contamination of mica surface by K_2_CO_3_, and shed new insight into electronic properties crucial for potential applications of 2D mica.

## Methods

The first-principles calculations were conducted using a periodic supercell model and employing the Vienna Ab-initio Simulation Package (VASP)^53^ with the Perdew–Burke–Ernzerhof (PBE) generalized gradient approximation (GGA) exchange—correlation functional^54^. A projector augmented wave (PAW) method^55,56^ was used as a plane wave basis set. For the plane-wave expansion, a 500 eV kinetic energy cutoff was set according to the cutoff energies testing with the energy error of 0.01 eV. The contribution of long range dispersion (van der Waals interaction) based on the DFT + D3 correction method of Grimme et al.^57^ was applied to all calculations. At least 15 Å vacuum is placed on both sides of the unit cell of all nanosheets to avoid images interaction in the presence of the periodic boundary condition.

The convergence criteria for the geometric optimization and energy calculation were set as follows: (1) self-consistent field energy tolerance is 1.0 × 10^−6^ eV, (2) all the atoms in the systems were fully relaxed and maximum force tolerance on each atom is smaller than 0.01 eV/Å. During the geometry optimization and the total energy calculations, the smearing value was set as 0.1 eV. A Monkhorst–Pack^58^ K-points mesh was used for sampling the Brillouin zone, where the number of K-points (N_K_) is changed to keep (N_K_ × L) with L being the lattice constant equal to ~ 30 Å and ~ 50 Å for structural relaxations and electronic calculations, respectively. The Bader charge was determined with the Bader scheme of charge density decomposition^59,60^.

## Data Availability

All data generated or analyzed during the current study are available from the corresponding author on reasonable request.
